# Bioluminescent Immunophage Sensors for the Quantification of Insulin

**DOI:** 10.1021/acsomega.5c08136

**Published:** 2026-01-28

**Authors:** Brian M. Miller, Brigette Wynne Q. Villamin, Vivian W. Liang, Bilge C. Yildiz, Teodora Nedic, Sanjana Sen, Elliot L. Botvinick, Gregory A. Weiss

**Affiliations:** † Department of Chemistry, 8788 University of California, Irvine, California 92697-2025, United States; ‡ Department of Pharmaceutical Sciences, 8788 University of California, Irvine, California 92697-3958, United States; § Department of Molecular Biology and Biochemistry, 8788 University of California, Irvine, California 92697-3900, United States; ∥ Department of Biomedical Engineering, 8788 University of California, Irvine, California 92697-2730, United States

## Abstract

Diagnosis and then therapeutic management of diabetes require accurate, rapid monitoring of key biomarkers. Currently, only glucose levels guide diabetes management. Reliance on one biomarker can lead to diabetes misdiagnosis and improper treatment. However, adding insulin to the diagnostic portfolio could improve patient outcomes. Toward this goal, we report BLIPS (Bioluminescent Immunophage Sensor), an easy-to-produce, point-of-care immunoassay platform for the detection and quantification of insulin. BLIPS combines the highly specific detection capabilities of antibodies, ease of handling and production of phage display, and a reliable, turn-on optical signal of nanoluciferase. Specifically, fragment antigen binding (Fab) regions of an antibody sandwich pair were each genetically fused to split-nanoluciferase fragments to detect insulin via the activity of the reconstituted nanoluciferase. These constructs are too insoluble for *E. coli* overexpression, but can be readily displayed on M13 phage. BLIPS allows for the detection of insulin down to 50 pM within minutes and provides a working range of up to 10 nM with no response to the competing and highly homologous peptide hormones IGF-1 and IGF-2. This work paves the way for rapid, low-cost bedside monitoring of insulin to improve the diagnosis and management of diabetes and also expands the generality of the robust split-luciferase sensor system to include phage display-solubilized receptors.

## Graphical abstract

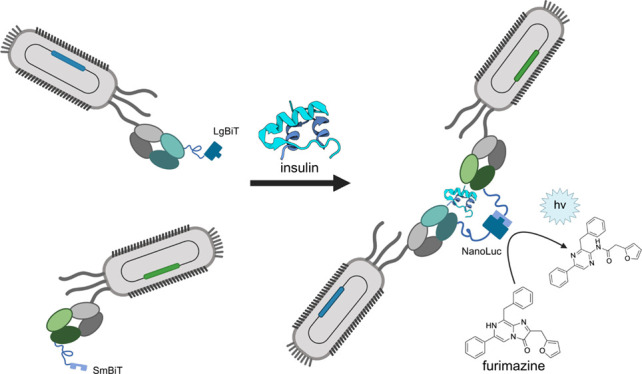

## Introduction

As of 2021, 38 million people, or roughly 10% of the population, suffer from diabetes in the United States.^1^ Furthermore, 1% to 2% of adults in the US have undiagnosed diabetes.^2^ Diabetes incidence can be largely divided into two categories: Type 1 and Type 2 diabetes (T1D and T2D, respectively). T1D comprises roughly 5% of diabetes cases, with incidences increasing by 2% to 5% annually.^3–5^ Diagnosis of T2D, the other 90% to 95% of cases, has been increasing significantly in adolescents.^1,6^ T1D results from an autoimmune disorder that attacks and destroys the insulin-producing β-islet cells of the pancreas.^7–9^ T1D individuals, therefore, must monitor their glucose levels to appropriately dose their treatment via injected insulin.^7^ T2D, however, is characterized by insulin resistance. T2D thus leads to an initial overproduction of insulin before insulin secretion is lost entirely.^10–12^

The mechanistic and treatment differences between T1D and T2D necessitate their accurate diagnosis. T1D and T2D diagnosis often occurs during visits to hospital emergency departments by patients experiencing severe hyperglycemia.^13–15^ Current methods used for diagnosing diabetes rely on assays of plasma glucose levels after fasting or 2 h post-meal. Assays for glycated proteins can also indicate high blood sugar levels associated with diabetes.^6,16^ These methods, however, monitor symptoms that overlap in T1D and T2D, causing a patient misclassification rate of 7% to 15%.^6,17^

Measurements of endogenous insulin levels can differentiate the diabetes type. High levels of insulin (hyperinsulinemia) are typically present in T2D patients, but little to no insulin is measured for individuals with T1D, which then guides proper treatments, including insulin therapy.^13–15,18^ C-peptide, a product of proinsulin maturation, provides an indirect method for quantifying insulin.^19,20^ As an indirect assay of insulin levels, C-peptide could inaccurately reflect current hormone levels due to differences in its stability compared to insulin. Insulin monitoring via the C-peptide is also important for the diagnosis of insulinoma and insulin autoimmune syndrome (IAS). Such hyperinsulinemia conditions arise from either islet cell tumors or an increased insulin half-life due to autoantibodies against insulin, respectively; both conditions result in severe hypoglycemia.^21–23^ Typical fasting levels of serum insulin for healthy individuals range from 25 to 70 pM; hyperinsulinemia patients have fasting concentrations of serum insulin >85 pM.^24–26^ We envision a rapid, point-of-care (POC) method to monitor insulin levels at the patient’s bedside in tandem with glucose monitors. This approach could dramatically improve differentiation between T1D, T2D, insulinoma, and IAS, along with consequent patient care (Table 1).

**Table 1:** A Diagnostic Portfolio Combining the Measurements of Glucose and Insulin

**Biomarker Measurement**		
**Glucose**	**Insulin**	**Diagnosis**
high	low to none	T1D
high	high	T2D
low	high	insulinoma or IAS

Patients known to have a risk for prediabetes and T2D could benefit from frequent monitoring of endogenous insulin levels for early diagnosis of insulin resistance. Hyperinsulinemia highly correlates with growing insulin resistance and often proceeds with significant changes in blood glucose levels.^27–30^ Early intervention during prediabetes increases the likelihood of preventing progression into T2D and improving survival.^31^ A POC insulin sensor could allow at-risk individuals to monitor their insulin levels away from the clinic and could aid in earlier intervention through preventative treatment.

Current methods for monitoring insulin or C-peptide levels consist of immunoassays, HPLC/MS-MS assays, and electrochemical biosensors utilizing aptamers or antibodies (Figure 1A).^32,33^ Gold-standard immunoassays, such as enzyme-linked immunosorbent assays (ELISA), or HPLC/MS-MS-based assays require sending patient serum or plasma samples to dedicated laboratories.^32,34^ This approach increases costs, limits sample rate, delays results, and subjects patients to invasive blood draws. These limitations thus encourage the development of decentralized POC sensors for insulin. To date, insulin POC sensors rely on electrochemical detection using antibodies and their derivatives, molecularly imprinted polymers, or aptamers for insulin-specific capture.^32,35–41^ Adaptation of electrochemical sensors can be hindered by their reliability and high-cost capture agents.^42^

**Figure 1:**
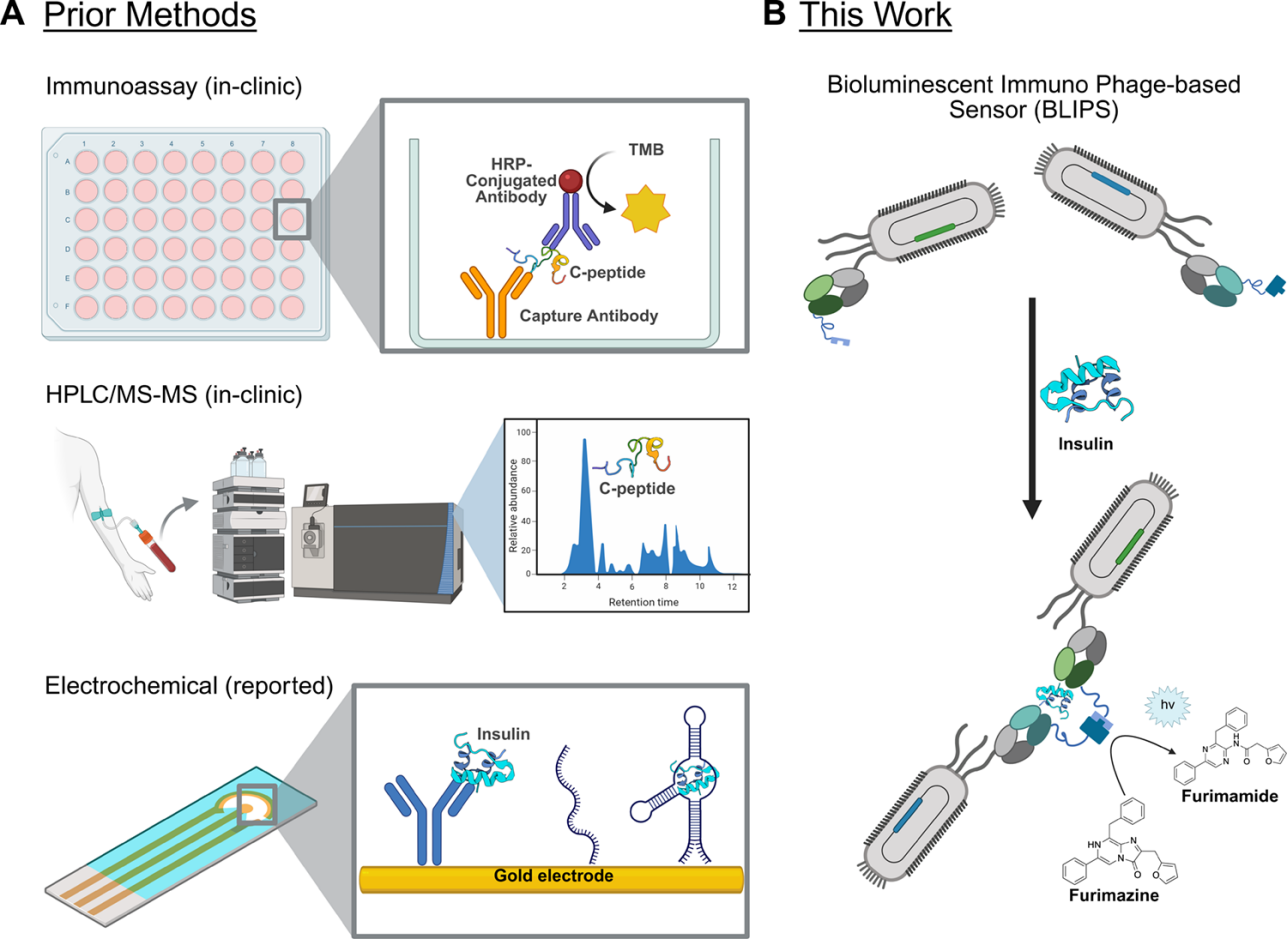
Methods for the quantification of insulin. (A) Immunoassays and HPLC/MS-MS-based assays are only viable in clinical laboratory settings due to their reliance on bulky equipment and skilled personel.^33^ Electrochemical sensors for at-home insulin monitoring have been reported,^35–41^ but the examples remain limited by cost and scale. (B) BLIPS presents a simpler approach to insulin quantification. BLIPS consists of two insulin binding, phage-displayed Fabs, each fused to half of split-NanoLuc (top). Sandwich binding to insulin drives the reconstitution of NanoLuc (bottom). The restored NanoLuc can then catalyze transformation of furimazine to furimamide, which produces light at λ_max_ = 460 nm. Created by Miller (2025). https://BioRender.com.

A direct, optically based sensor for insulin could overcome many challenges currently facing insulin quantification. Generally, optical-based sensing offers high sensitivity and reusability, facilitates reduction in device size, and has reduced costs.^43,44^ Successful optical-based sensors have been developed using Förster resonance energy transfer, bioluminescence resonance energy transfer, and surface-enhanced Raman scattering.^44–46^ Recently, sensors using the reconstitution of a split-nanoluciferase (NanoLuc) allow sensitive, low background metabolite detection.^47–49^ NanoLuc-based quantification offers improvements over other optical methods due to its avoidance of an external excitation source, high signal-to-noise ratios, and enhanced stability. Thus, the approach offers versatile molecular recognition possibilities to target a range of biomolecules, including RNA and proteins.^50–53^

This report describes the development of a bioluminescent immunophage sensor (BLIPS) as an easy-to-produce sensor for the detection and quantification of insulin (Figure 1B). This approach uses two complementary insulin-specific fragment antigen binding (Fab) regions displayed on the M13 bacteriophage; each phage-displayed Fab is also fused to either the large subunit (LgBiT, 18 kDa) or the small subunit (SmBiT, 1.3 kDa) of split-NanoLuc. Formation of the Fab-insulin sandwich complex reconstitutes NanoLuc, providing a quantifiable optical signal. Fabs offer improved properties over full antibodies due to their smaller sizes, reduced costs, and easier engineering.^54^

This strategy required two Fabs that could noncompetitively bind insulin as a sandwich pair. Here, we turned to the Fab domains of the insulin-binding antibodies HUI-018, termed HUI, and OXI-005, termed OXI.^55^ These Fabs bind different epitopes within insulin and form an insulin binding sandwich pair. For the production of these Fabs, we utilized the display on coat protein 3 (P3) of filamentous M13 phage.^56^ Phage display can aid in the bacterial production of difficult-to-solubilize proteins and provides a direct link between the Fab and its encoding gene.^57–60^ In addition, phage display constructs offer a viable platform for direct biomarker detection.^61^

Development of BLIPS required several phases. First, the system was constructed and characterized (Figure 2). After binding to insulin was confirmed, in vitro assays were performed to characterize the BLIPS behavior and sensitivity (Figure 3). Further assays quantified the behavior of BLIPS in synthetic urine (Figure 4) and with other insulin-like hormones to determine specificity (Figure 5). The results presented here demonstrate the potential for BLIPS to provide an effective POC sensor.

**Figure 2:**
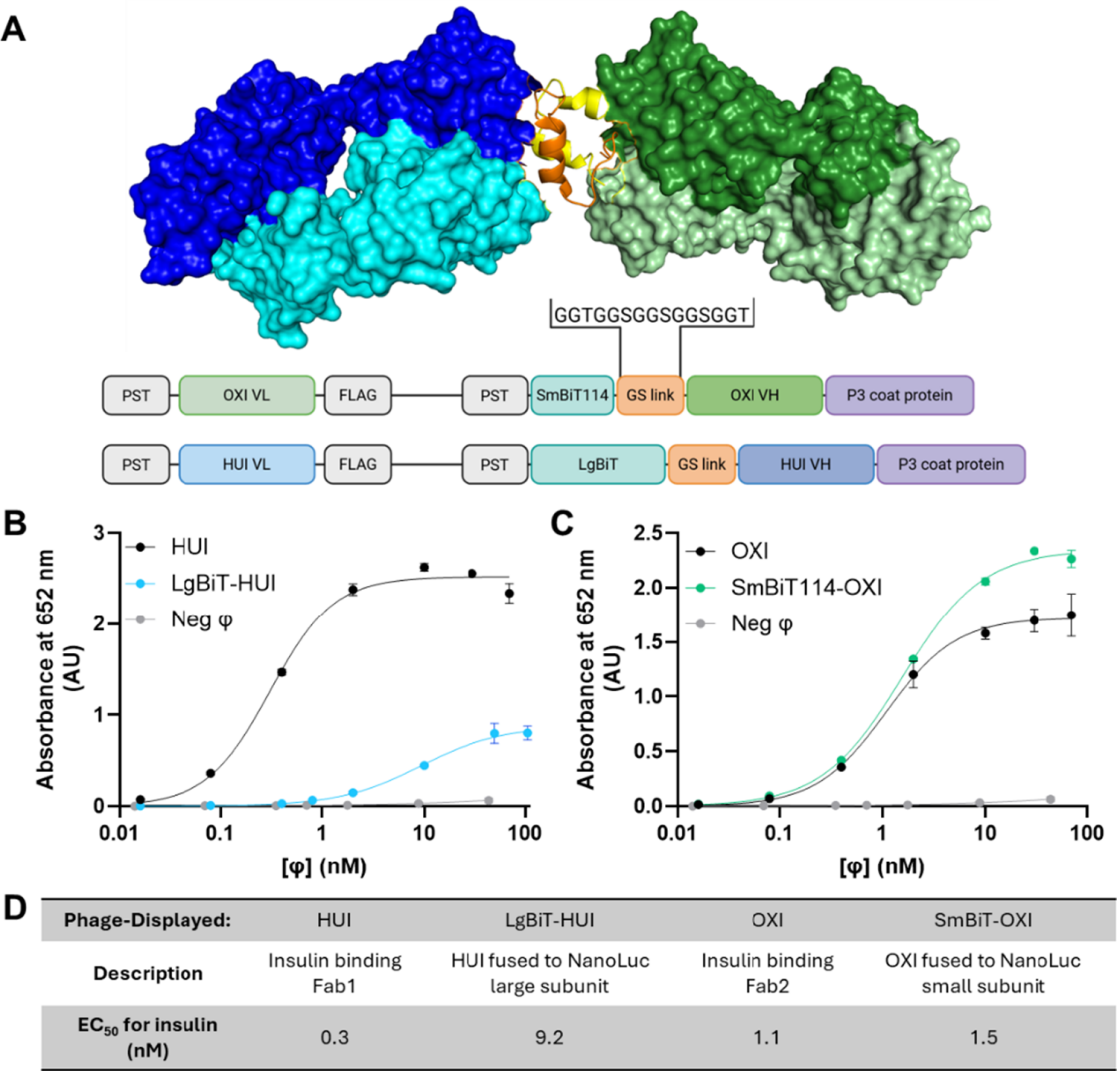
Binding to insulin by NanoLuc-Fab fusions. (A) Using a computational model of the HUI and OXI insulin sandwich complex, distances between the N-termini of the heavy chains for each Fab were estimated to design the linker required for luciferase complementation. Crystal structures of HUI (variable light chain, VL, in light blue, variable heavy chain, VH, in dark blue, PDB 6Z7W), the OXI (VL in light green, VH in dark green, PDB 6Z7Y), and insulin (A chain in yellow, B chain in orange, PBD 6Z7Y) were modeled using PyMOL. The flexible Gly-Ser linker in parentheses was used for each construct. PST indicates the periplasmic signal peptide. The dose-dependent indirect phage ELISAs of (B) HUI and (C) OXI assessed binding to insulin with and without fusion to the NanoLuc system. Phage concentration is indicated as [ϕ]. (D) EC_50_ values were calculated using curve fits to the data from indirect phage ELISAs. Negative control phage (Neg ϕ) lacked displayed proteins, and no EC_50_ was observed. Throughout this report, error bars indicate the standard error for technical replicates (*n* = 3); each data point includes error bars, although some are too small to be visible.

**Figure 3:**
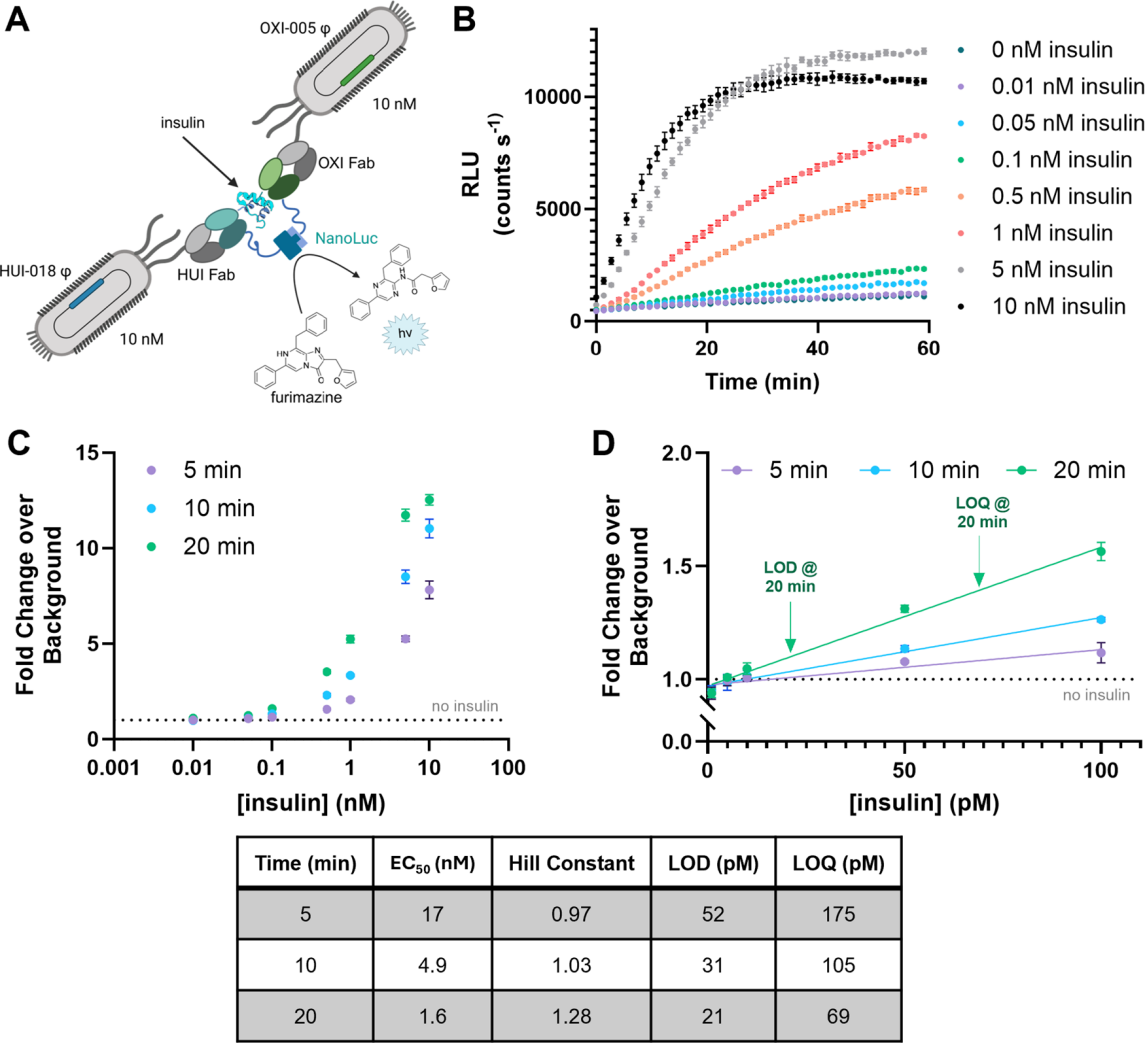
Characterization of insulin detection by BLIPS. (A) Fusions of either the HUI Fab and LgBiT, or the OXI Fab and SmBiT114 were displayed on P3. BLIPS consisted of 10 nM of each split-NanoLuc-Fab fusion. Reactions were initiated upon the addition of the indicated concentrations of insulin and 10 μM furimazine substrate. The emission was recorded for 1 h at room temperature. (B) The luminescence signal for BLIPS at varying concentrations of insulin was monitored over time. (C) Data were analyzed as a fold change in bioluminescent data over the no insulin negative control (dashed line) after incubation periods of 5, 10, and 20 min. Data were fit to a Hill equation to determine the sensor’s working range. (D) Sensor behavior at low concentrations of insulin was plotted using linear regression at different incubation times. The limit of detection (LOD) was calculated as the concentration at 3 times the standard error of the no insulin control (3σ) and the limit of quantification (LOQ) at 10 times the standard error (10σ). Hill constant, EC_50_, LOD, and LOQ show increasing sensitivity over time, as expected.

**Figure 4:**
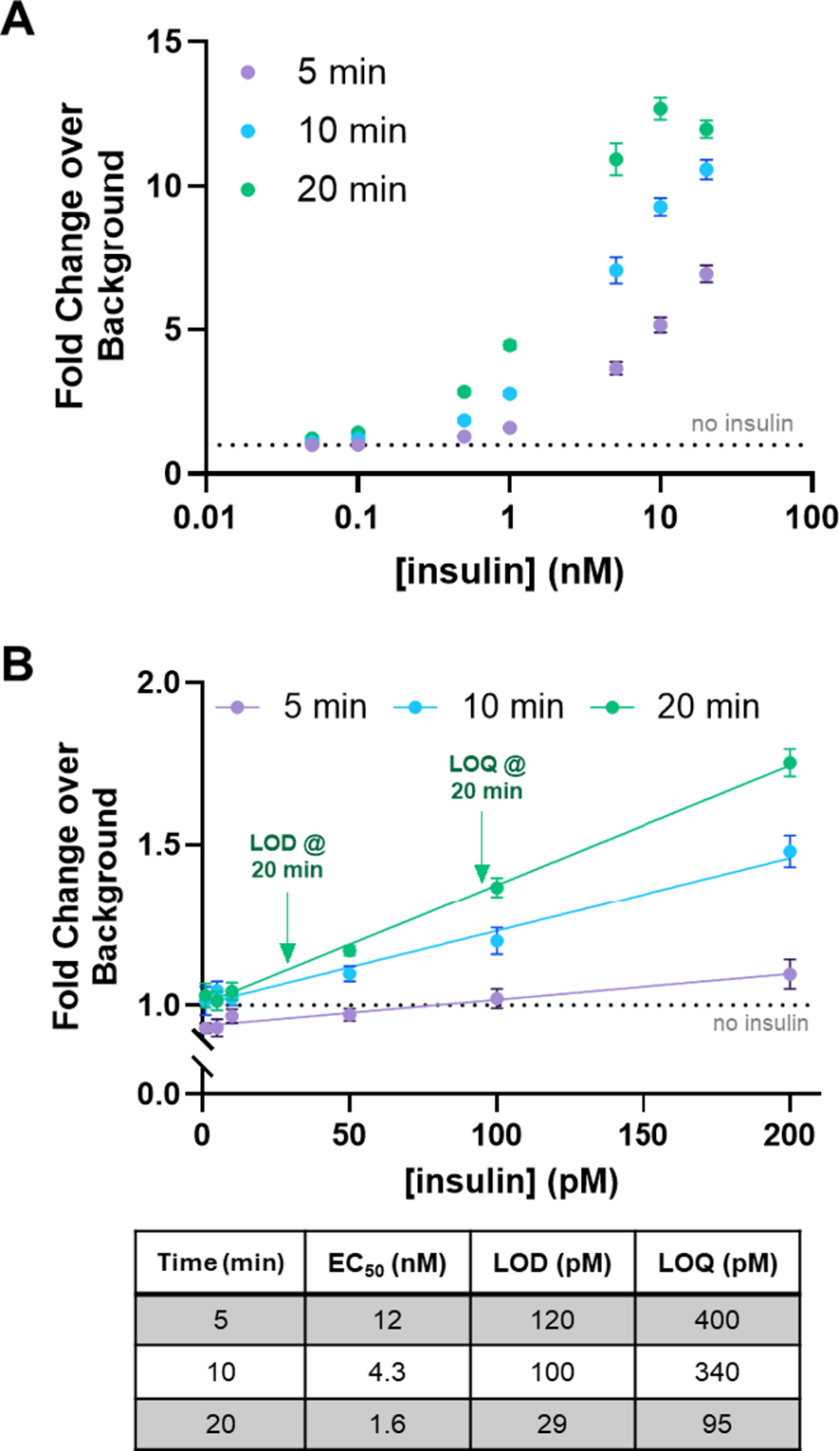
Investigation of BLIPS in synthetic urine. (A) BLIPS efficacy was tested in 50% synthetic urine. Data were analyzed as a fold change in bioluminescent data over the no insulin negative control after incubation periods of 5, 10, and 20 min. (B) The sensor behavior at low concentrations of insulin was investigated to determine LOD and LOQ. LOD and LOQ were calculated as described in Figure 3D.

**Figure 5:**
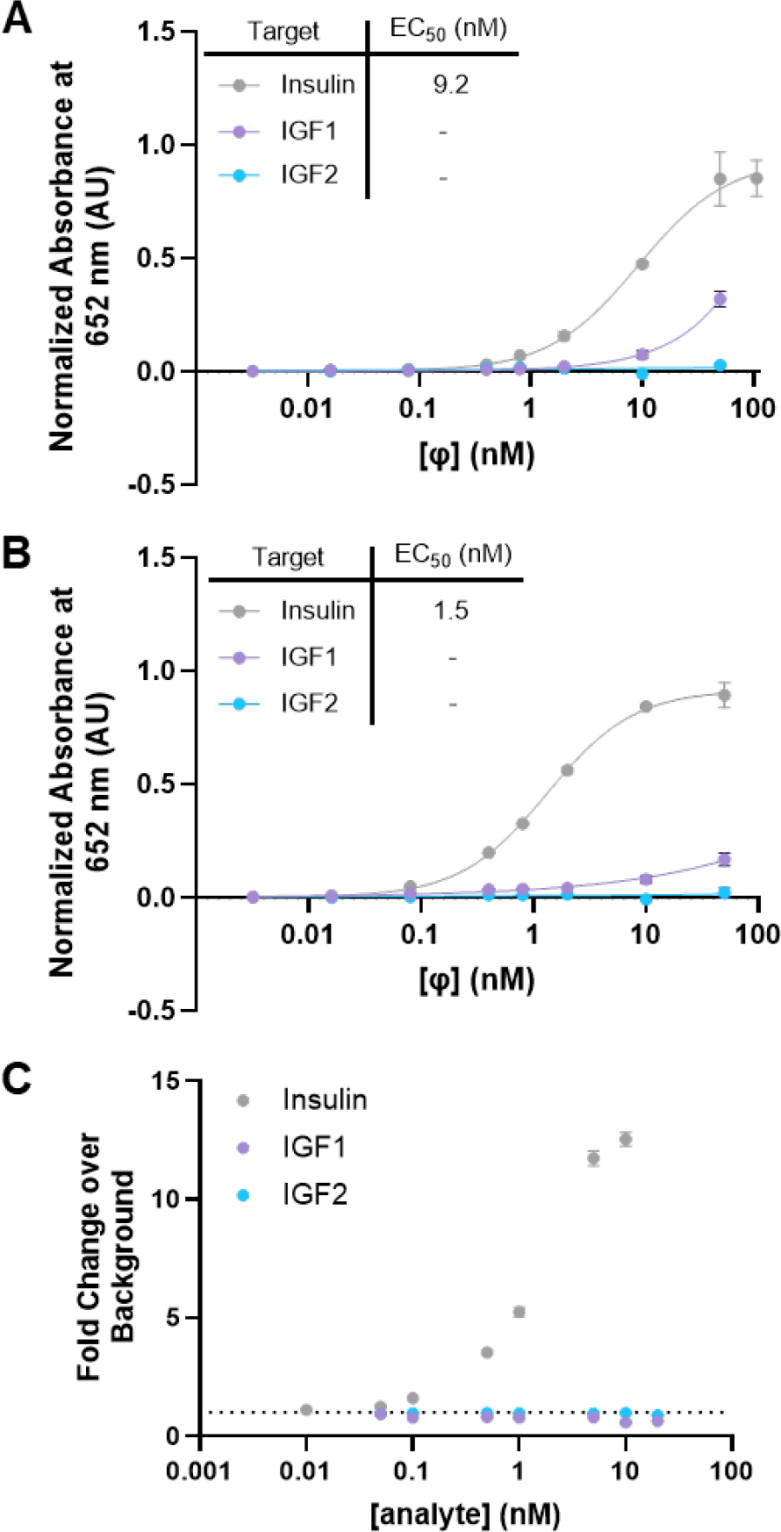
Cross-reactivity with insulin-related hormones investigated by ELISA and BLIPS. Dose-dependent, indirect phage ELISAs of LgBiT-HUI (A) and SmBiT-OXI (B) binding to IGF1 and IGF2 were compared under identical conditions for binding to insulin. Data was normalized to the insulin signal. Dash (−) indicates EC_50_ values that were unable to be calculated. (C) The fold-change, dose–response curves for BLIPS with IGF1 and IGF2 were assessed by using identical conditions for binding to insulin after a 20 min incubation period. BLIPS activity was not observed for IGF1 or IGF2 at any concentration.

## Results and Discussion

### Sensor Design and Analysis of Phage Constructs

The split-NanoLuc system and insulin-binding Fab sandwich described above were combined into an insulin-sensitive, luminescence-based sensor. Specifically, an open reading frame (ORF) was constructed to encode codon-optimized LgBiT and SmBiT fused to the N-terminus of the heavy chains of HUI and OXI, respectively. Structural analysis and computational modeling guided the design of the LgBiT-HUI and SmBiT-OXI constructs. Structures of insulin bound to HUI and OXI (PDB ID 6Z7W and 6Z7Y, respectively)^55^ were visualized using PyMOL.^62^ This modeling enabled the estimation of the linker length for each split-NanoLuc-Fab fusion (Figure 2A). A flexible glycine-serine [GG­(S/T)]_5_ linker for each construct was chosen to maximize the potential formation of the split enzyme complex. Adding to evidence that phage display dramatically enhances protein solubility and *E. coli* overexpression levels,^60,63^ only split-NanoLuc-Fab fusions displayed on the phage surface could be produced in high yield. No products were isolated from attempts at conventional protein overexpression of the split-NanoLuc fusions in *E. coli* (Figure S1A).

We investigated two variants of SmBiT (variant 99 and variant 114) to identify the NanoLuc pair with the highest sensitivity and lowest background. The two SmBiT variants have a 1000-fold difference in their *K*_d_ for LgBiT.^48^ The higher affinity SmBiT99 fusion produced a significantly higher background and less sensitivity in signal over background upon the addition of insulin, which triggers binding to LgBiT-HUI (Figure S2A). This suggests that the affinity of SmBiT99 for LgBiT overpowers the sandwich complementation of the Fabs affinity for insulin. Therefore, we chose SmBiT114 for its minimal background luminescence and better signal-to-noise ratio upon the addition of insulin (Figure S2B). Hereafter, the resulting fusions are termed LgBiT-HUI and SmBiT-OXI, in place of SmBiT114-OXI.

The proximity of the NanoLuc fragments to the binding epitopes of the Fabs could potentially interfere with the binding of HUI and OXI to insulin. Binding of the individual Fabs, LgBiT-HUI and SmBiT-OXI, to insulin was assessed using an indirect-phage ELISA; this assay measures the binding between microtiter plate-coated insulin and the phage-displayed Fabs using an antiphage, HRP-conjugated antibody. LgBiT caused a significant increase in the EC_50_ values of HUI for insulin, with an EC_50_ of 0.3 nM for HUI and 10 nM for LgBiT-HUI (Figure 2B). The presence of SmBiT, however, had less impact on insulin binding by OXI, with an EC_50_ of 1.0 nM for the OXI and 1.5 nM for the SmBiT-OXI complex (Figure 2C). The impact of NanoLuc fragments on insulin binding appears to be dependent on fragment size. LgBiT has a significant deleterious effect on HUI EC_50_, but the much smaller SmBiT has little to no impact on the binding of OXI to insulin. To address this, the linker connecting LgBiT to HUI can be further optimized. Specifically, we envision future investigations to examine the relationship between linker length and rigidity on HUI sensitivity to insulin and on the efficacy of split-luciferase reconstitution.

### Evaluation of BLIPS with Insulin

The next experiments evaluated BLIPS sensitivity for insulin measurements in solution, including measurement speed, limit of detection, limit of quantification, and specificity. Each assay included purified phage displaying the split-NanoLuc-Fab fusions at various concentrations, along with insulin also at various concentrations, and furimazine (10 μM). After simultaneous addition of insulin and furimazine, the luminescence signal was monitored at room temperature (Figure 3A). For sandwich binding complexes, the affinities of each receptor for the target can strongly impact sensor response and sensitivity.^50^ Additionally, too high a concentration of either the LgBiT or SmBiT components can increase background complementation and decrease the signal-to-noise ratio.

Different concentrations of LgBiT-HUI and SmBiT-OXI were assessed (Figure S3). This optimization experiment quickly yielded improvements to BLIPS sensor performance. Here, a >2-fold improvement in signal over background resulted from doubling LgBiT-HUI phage concentration to 10 nM. The concentration of SmBiT-OXI phage had little to no effect on signal over background. Higher precision and smaller error resulted from equal concentrations of each BLIPS component. Thus, all subsequent BLIPS experiments used 10 nM concentrations of the two split-NanoLuc-Fab fusion proteins.

Next, we investigated the BLIPS’ sensitivity for insulin over a range of insulin concentrations and assay times (Figure 3B). The rate of furimazine turnover and thus luminescence directly correlates with the concentration of insulin. Peak luminescence occurred within 20 min at 10 nM insulin (Figure 3B). At higher concentrations of insulin (≥10 nM) well above physiological concentrations, autoinhibition of the signal, sometimes termed a hook effect,^64^ was observed. At insulin concentrations equal to or above the phage concentration, individual copies of insulin can bind to each Fab separately, thus inhibiting sandwich complex formation and the subsequent reconstitution of the split-NanoLuc enzyme.

Further characterization examined the background correction and measurement times. Background luminescence was observed from the inherent binding affinity of LgBiT for SmBiT. To account for this, luminescence output at various time points was analyzed as a fold-change over background based on the signal for the negative, no insulin control (Figure 3C). The sensor response was fit to a Hill equation (Figure S4).^65^ Comparing values measured for EC_50_ and Hill constants after 3 time periods (5, 10, and 20 min) revealed interesting trends (Figure 3). First, the measured EC_50_ values decreased over time. Second, the measured Hill constant demonstrated some cooperativity for complex formation, which increased over time. Thus, increasing the incubation time can improve the sensitivity of this luciferase-based sensor system.

The lowest insulin detection capabilities of BLIPS were next quantified. The limit of detection (LOD) and limit of quantification (LOQ) were determined using the 3σ and 10σ rule (n x standard error of the blank divided by the slope of the calibration curve), respectively.^66–70^ As described above, these parameters were found to be time sensitive. LOD stabilized after 10 min at ∼30 pM (Figure 3D). This suggests BLIPS, within 10 min, can detect insulin within the normal fasting insulin range for nondiabetics (25–70 pM).^24^ Furthermore, 5 min will be sufficient to establish hyperinsulinemia in most patients, as prior work suggests fasting insulin levels of >85 pM as an appropriate cutoff for hyperinsulinemia.^25,26^

### Insulin Sensing in Physiological Fluids

POC sensors must monitor biomarker concentrations directly in patient fluids. To model measurements in physiological fluids, BLIPS was assessed in synthetic urine and porcine serum, to which insulin was added. BLIPS measurements in 100% synthetic urine resulted in a reduction in sensitivity (Figure S5A). Specifically, the LOD and LOQ increased roughly 6- to 7-fold at all time points compared with measurements in PBS (Figure S5B). Diluting the synthetic urine to 50% v/v in PBS recovered the sensor’s sensitivity (Figure 4A). Here, when fit to the Hill equation, EC_50_ values were analogous to those observed in PBS. The LOD and LOQ, similarly, recovered to levels close to those observed in PBS (Figure 4B). The decrease in sensitivity observed for 100% synthetic urine could be due to the presence of urea, which can disrupt intraprotein interactions and protein folding.^71^ Preliminary investigations of BLIPS sensitivity in serum showed some efficacy after the serum was diluted to 5% (v/v) in PBS (Figure S6). These results illustrate the challenges to solve before using BLIPS in clinical settings. First, engineering Fabs for improved binding to insulin can recover lost sensitivity at the described dilutions. Second, optimization with other SmBiT variants can create a brighter signal that is less impacted by the background associated with furimazine autoxidation and signal suppression from absorbance in biological matrices.^48^

### Evaluation of BLIPS with Competing Hormones

The specificity of sensors for their target of interest is a major factor in clinical usefulness. To this end, BLIPS must be selective for insulin and reject other proteins, including close homologues. Insulin-like growth factors 1 and 2 (IGF1 and IGF2) have roughly 50% and 47% amino acid homology to insulin; the three are known to readily bind to each other’s receptors.^72–77^ Both IGFs have important roles in growth and development.^77^ In the case of diabetes, abnormal levels of IGF1 and overexpression of IGF2 have been implicated in the development of insulin resistance and T2D.^72,75^ Serum concentrations are approximately 50–125 pM for IGF1 and 200 pM for IGF2, which is within the range of concentrations required for insulin sensing.^78^ Therefore, ensuring that IGF1 and IGF2 do not interfere with insulin monitoring is vital for accurate measurements.

Fab binding to each IGF was assessed via indirect phage ELISA (Figure 5A, B) and BLIPS (Figures 5C and S7). Through ELISA, some binding to IGF1 by LgBiT-HUI was observed, but only at IGF1 concentrations >100-fold higher than physiologically relevant concentrations. No affinity of LgBiT-HUI to IGF2 was observed. SmBiT-OXI had no observable affinity for both IGF1 and IGF2. Additionally, BLIPS yielded no discernible luminescence signal at increasing concentrations of both IGF1 and IGF2. Thus, we conclude that no sandwich binding occurs for IGF1 or IGF2. The results illustrate the power and specificity of the BLIPS system.

## Conclusions

We report BLIPS, an optical-based POC sensor, designed for the sensitive detection of insulin. We demonstrate that BLIPS provides robust, highly sensitive, and selective detection of insulin while being readily produced. Such capabilities result from (1) the solubilization and consequent ease of *E. coli* production afforded by phage display, (2) the high affinity and specificity of antibody sandwich binding, and (3) the robust and noiseless optical signal of the split-NanoLuc system. We observed low picomolar sensitivity, high selectivity over insulin-like growth factors, and good tolerance to biological denaturants. Accurate measurements of insulin levels are vital in diabetes diagnosis and monitoring of insulin-resistance progression. The aforementioned capabilities could allow BLIPS to aid in diabetes disease management and thus improve patient outcomes.

## Materials and Methods

### Materials

Unless otherwise specified, reagents were sourced from Sigma-Aldrich. Q5 DNA polymerase, HiFi DNA Assembly Master Mix, KLD (kinase, ligase, and DPN1) Master Mix, and relevant buffers were purchased from New England Biolabs. Recombinant human insulin was sourced from MP Biomedicals. Primers were purchased from Integrated DNA Technologies. Genes encoding HUI-018 and OXI-005 were ordered from Twist Biosciences. Plasmids were sequenced by a Plasmidasaurus. The vectors containing the genes of full NanoLuc and NanoLuc fragments were generous gifts from Professor Jennifer Prescher of the University of California, Irvine (UCI).

### Cloning

For the phagemid-based display of Fabs fused to P3, the modified pS1602 phagemid was used. First, Fab-encoding DNA sequences were cloned into the appropriate phagemid location using a Gibson assembly (New England Biolabs). Two PCRs were performed with the phagemid or pTwist (Twist Biosciences) containing the Fab gene to generate the vectors and inserts, respectively. Fragments were assembled per the manufacturer’s instructions. Assemblies were transformed into DH5α *E. coli* competent cells, and transformants were plated on a carbenicillin (CARB) supplemented (50 μg/mL) agar plate before incubation at 37 °C overnight. Colonies were grown in seed cultures in 5 mL of Luria–Bertani broth supplemented with 50 μg/mL CARB before phagemid DNA was isolated using the QIAprep spin miniprep kit according to the manufacturer’s instructions. Next, NanoLuc fragments were cloned onto their respective Fabs using Gibson Assembly (LgBiT) or Q5 site-directed mutagenesis (SmBiT114) per the manufacturer’s instructions and processed as described above. Lastly, the flexible linker was cloned using Q5 site-directed mutagenesis per the manufacturer’s instructions and processed as described above (Figures S8, S9 and Table S1).

### Phage Propagation

Phagemid DNA was transformed into SS320 chemically competent *E. coli* cells, and cells were plated on LB agar plates supplemented with 50 μg/mL CARB before incubation overnight at 37 °C. A single colony was inoculated into 15 mL of 2YT (autoclaved solution of 1.6% w/v tryptone, 0.5% w/v NaCl, and 1% w/v yeast extract in water) supplemented with 50 μg/mL CARB and 2.5 μg/mL tetracycline. The culture was grown at 37 °C with shaking until its OD_600_ reached 0.55 to 0.65. Next, IPTG was added to a final concentration of 30 μM, and sufficient M13KO7 was added to achieve a multiplicity of infection of 4.6. The culture was incubated for an additional 45 min before 8 mL of culture was used to inoculate 300 mL of 2YT supplemented with 50 μg/mL CARB, 20 μg/mL kanamycin (KAN), and 30 μM IPTG. This culture was incubated at 30 °C while being shaken for 18 h.

The culture was centrifuged at 10 krpm (15,300 *g*) for 10 min. The supernatant was transferred to a fresh centrifuge tube containing a mixture of 20% w/v PEG8000 and 2.5 M NaCl at 1/5 of the supernatant’s volume. The tube was mixed and incubated on ice for 30 min. The solution was centrifuged at 10 krpm (15,300 *g*) for 15 min. The supernatant was removed, and the pellets were resuspended in resuspension buffer (PBS pH 8, 10% v/v glycerol, and 0.05% v/v Tween20) and centrifuged again at 10 krpm (15,300*g*) for 4 min to pellet insoluble debris. Aliquots (1 mL) were then flash frozen and stored at −80 °C. When needed, aliquots were thawed on ice before the addition of 200 μL of a mixture of 20% (w/v) PEG8000 and 2.5 M NaCl. Aliquots were mixed and incubated on ice for 30 min. Solutions were centrifuged at 13,000*g,* and pellets resuspended in PBST (PBS pH 7.5 for NanoLuc assays, pH 8 for ELISAs, 0.05% v/v Tween20). Aliquots were centrifuged again at 13,000 *g* for 4 min to pellet insoluble debris. Supernatants were combined, and phage concentrations were quantified by measuring the solution’s absorbance at 268 nm.

### Indirect Phage ELISAs

A Nunc Maxisorp 96-well plate was coated with 100 μL of 10 μg/mL of antigen (insulin, IGF1, IGF2, or BSA) in 50 mM Na_2_CO_3_ at pH 9.6 and incubated overnight at 4 °C. The coating solution was discarded, and the plate was blocked with 300 μL of blocking buffer (0.2% BSA in PBS at pH 8) for 1 h. The blocking solution was discarded, and the wells were washed three times with PBST pH 8. The plate was then incubated with 100 μL of serially diluted phage in binding buffer (PBST pH 8 with 0.2% BSA) for 1 h. Phage solutions were discarded, and the wells were washed five times with PBST pH 8. Next, 100 μL of 1:5000 diluted Anti-M13 Monoclonal Antibody conjugated to HRP (Creative Diagnostics) in binding buffer was added to the plate and incubated for 30 min. The wells were washed five times with PBST pH 8 and once with PBS pH 8. To the plate, 100 μL of 1-Step Ultra TMB-ELISA Substrate Solution (ThermoFisher) was added. After sufficient signal had developed, absorbance at 652 nm was measured with an Epoch Microplate Spectrophotometer (BioTek), and the resulting data were analyzed and fit using GraphPad Prism 10.

### Luminescent Assays

Phages displaying HUI and OXI were diluted to 40 nM in PBST pH 7.5 or synthetic urine (Ricca Chemical). To a black 96-well plate, 25 μL of each phage solution was added. Serially diluted antigen in buffer or biological media was combined with a furimazine substrate (Promega, Nano-Glo) to a final concentration of 20 μM furimazine and 2× the desired concentration of antigen. Reactions were initiated upon the addition of 50 μL of the antigen-furimidin mixture to the plate. Luminescence was measured with a luminometer (Tecan) for 1 h. The resulting data were analyzed, and curve fits were generated using GraphPad Prism 10.

## Supplementary Material


