# Supplementary material for: Bioluminescent Immunophage Sensors for the Quantification of Insulin
Source: ACS Omega. 2026 Jan 28;11(5):7367–76. doi: 10.1021/acsomega.5c08136 (PMC12902864; doi:10.1021/acsomega.5c08136)
Supplement: Supplementary file 1 [file ao5c08136_si_001.pdf]

Supporting Information

Bioluminescent Immunophage Sensors for Quantification of Insulin

Brian M. Miller[1], Brigette Wynne Q. Villamin[1], Vivian W. Liang[1], Bilge C. Yildiz[1], Teodora Nedic[2], Sanjana Sen[3], Elliot L. Botvinick[4*], and Gregory A. Weiss[1,2,3,*]

1. Department of Chemistry, University of California, Irvine, CA 92697-2025, USA

2. Department of Pharmaceutical Sciences, University of California, Irvine, CA 92697-3958, USA

3. Department of Molecular Biology and Biochemistry, University of California, Irvine, CA 92697-3900, USA

4. Department of Biomedical Engineering, University of California, Irvine, CA 92697-2730, USA

Table of Contents

Figure S1: Unsuccessful overexpression of Fabs not displayed on phage. S2

Figure S2: Comparison of two SmBiT variants for BLIPS assays. S2

Figure S3: Optimization of phage concentration for BLIPS assays of insulin concentration. S3

Figure S4: Characterization of BLIPS binding to insulin. S3

Figure S5: Further investigations of BLIPS in synthetic urine. S4

Figure S6: Preliminary investigation of BLIPS in porcine serum. S4

Figure S7: BLIPS with insulin-like hormones. S5

Figure S8: DNA sequence of LgBiT-HUI phage. S6

Figure S9: DNA sequence of SmBiT114-OXI phage. S7

Table S1: List of DNA oligos used in cloning procedures. S8

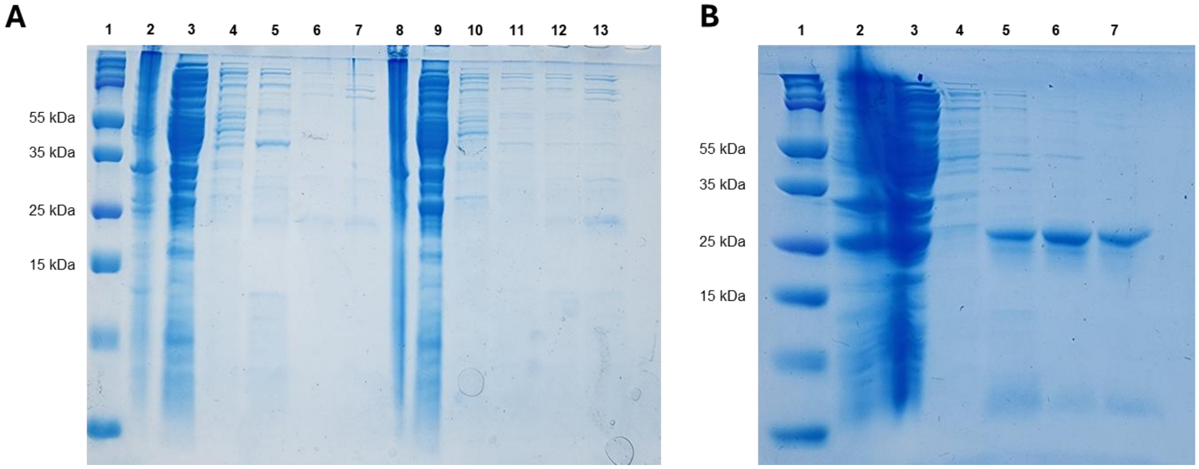

Figure S1: Unsuccessful overexpression of Fabs not displayed on phage. SDS-PAGE (12% acrylamide) of Fabs overexpressed off the phage surface. **(A)** HUI cell pellet (lane 2), lysate (lane 3), and IMAC purification fractions using a gradient of imidazole concentrations from 0 to 250 mM (lanes 4-7), OXI cell pellet (lane 8), lysate (lane 9), and IMAC purification fractions using a gradient of imidazole concentrations from 0 to 250 mM (lanes 10-13). **(B)** An *E. coli* optimized Herceptin Fab (HER2) was expressed off phage as a positive control. HER2 was expressed in the same vector used for overexpression of HUI and OXI in panel A. Here, we present the Fab cell pellet (lane 2), lysate (lane 3), and IMAC purification fractions using a gradient of imidazole concentrations from 0-250 mM (lanes 4-7). The PAGE Ruler Plus Prestained Protein Ladder (Thermo) was used to estimate protein sizes (Lane 1 in panels A and B).

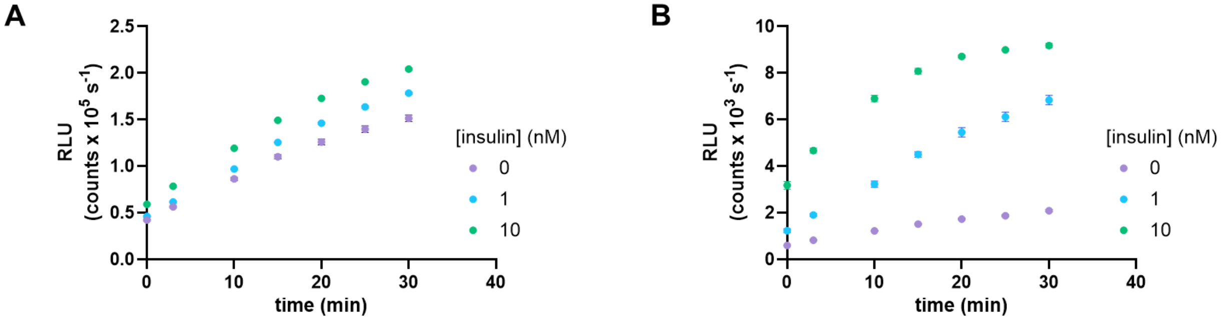

Figure S2: Comparison of two SmBiT variants for BLIPS assays. BLIPS binding to insulin was assayed with two different variants of SmBiT: **(A)** SmBiT99-OXI phage or **(B)** SmBiT114-OXI phage. Throughout this report, error bars for all graphs indicate standard error of technical replicates (n = 3); each data point includes error bars, though some are too small to appear.

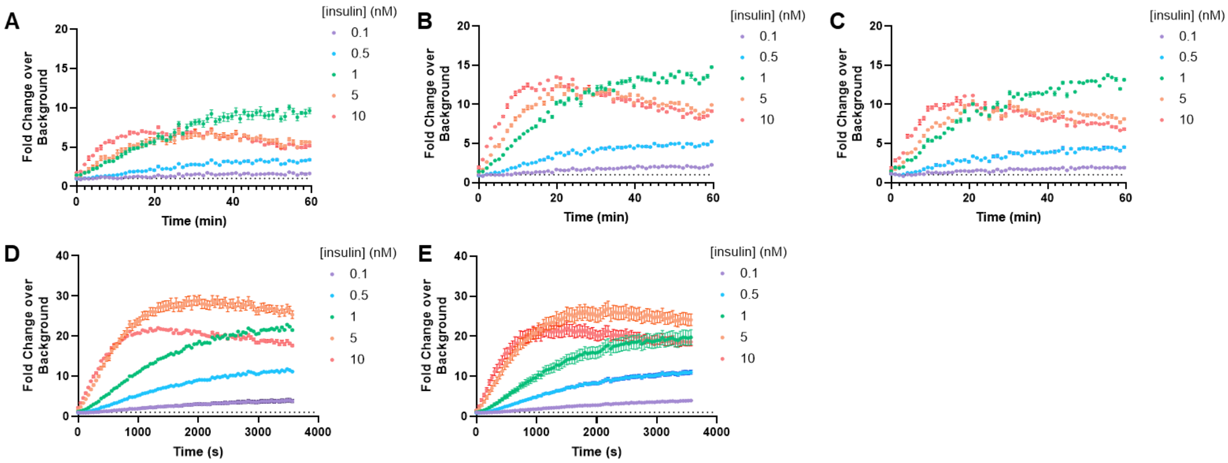

Figure S3: Optimization of phage concentrations for BLIPS assays of insulin concentration. The following conditions were tested: **(A)** 5 nM LgBiT-HUI + 5 nM SmBiT-OXI, **(B)** 5 nM LgBiT-HUI + 10 nM SmBiT-OXI, **(C)** 5 nM LgBiT-HUI + 20 nM SmBiT-OXI, **(D)** 10 nM LgBiT-HUI + 10 nM SmBiT-OXI, **(E)** 10 nM LgBiT-HUI + 20 nM SmBiT-OXI.

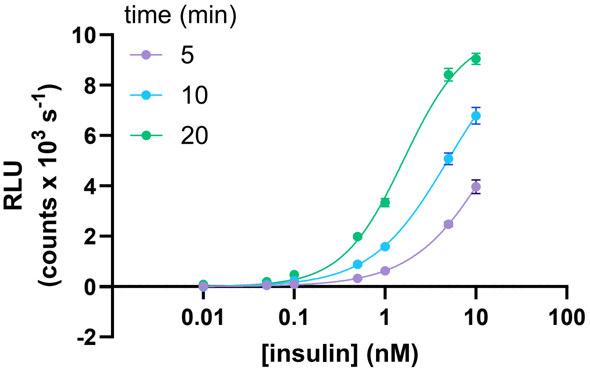

Figure S4: Characterization of BLIPS binding to insulin. In this experiment, the background was subtracted from the luminescence of BLIPS at each concentration of insulin. Data was collected at the indicated times and fit to the Hill Equation.

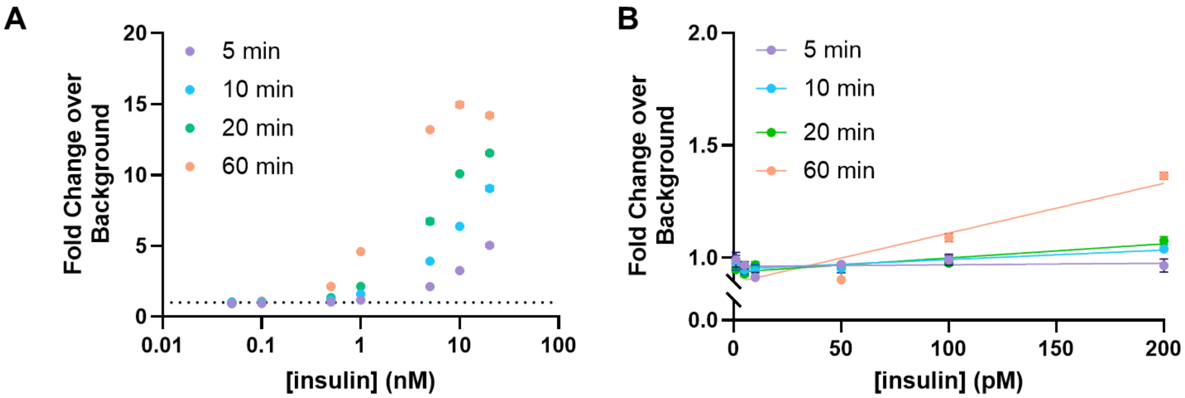

Time (min)	EC_{50} (nM)	LOD (pM)	LOQ (pM)
5	23	430	1400
10	13	130	420
20	5	100	340
60	2	90	290

Figure S5: Further investigations of BLIPS in synthetic urine. A) BLIPS efficacy was tested in 100% synthetic urine. Data was analyzed as a fold change in bioluminescent data over the no insulin negative control after incubation periods of 5, 10, 20, and 60 min. **B)** The sensor behavior at low concentrations of insulin was plotted using linear regression at different incubation times. The limit of detection (LOD) was calculated as the concentration at 3 times the standard error of the no insulin control (3σ), and the limit of quantification (LOQ) at 10 times the standard error (10σ).

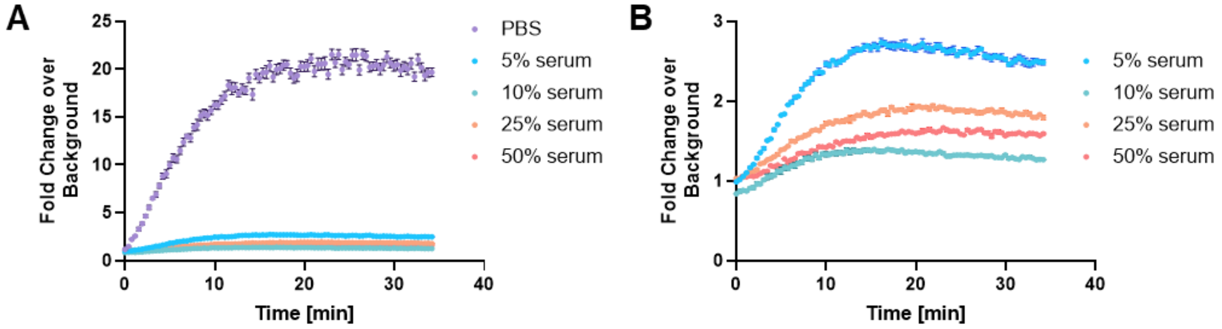

Figure S6: Preliminary investigation of BLIPS in porcine serum. A) BLIPS binding to 10 nM insulin was assayed in varying v/v% serum and compared to PBS. **B)** If PBS is removed from the graph, a trend becomes visible between different v/v% serum. Sensitivity to insulin recovers in 5% v/v serum. Further engineering can recover BLIPS in serum to the sensitivity observed in PBS.

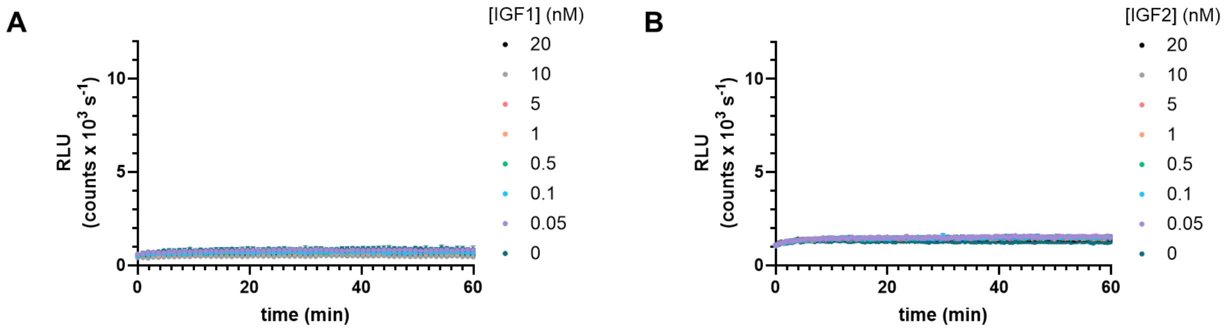

Figure S7: BLIPS with insulin-like hormones. BLIPS binding to **(A)** IFG1 or **(B)** IGF2 was assayed. Essentially no sandwich binding activity of the BLIPS assay was observed for these two hormones, which are closely related to insulin.

atgaaaaagaatatcgcatttcttcttgcatctatgttcgtttttctattgctacaaatgcctatgcaGACATTGTAATGACCCAGTCTCAA
AAATTTATGAGCACCTCAGTCGGTGACCGCGTAAGCATTACTTGTAAAGCGTCACAAAATGTA
CGGACAGCTGTTGCCTGGTATCAACAGCGACCAGGCCAATCACCGAAAGCGTTAATTTATCTG
GCCTCGAACCGTCATACGGGAGTTCCCGATCGTTTCACCGGTTCCGGGAGTGGCACCGATTTT
ACGCTTACAATCACTAATGTGCAGTCTGAGGACCTCGCAGATTACTTTTGCTTACAGCATTGGA
ACTATCCCTTAACCTTTGGCAGTGGTACAAAACTGGAAATTAAGAGAGCCGATGCGGCGCCG
ACCGTATCTATTTTTCCGCCTTCTAGCGAACAGCTCACAAGCGGTGGAGCAAGCGTGGTTTGT
TTCCTCAACAATTTTTATCCGAAAGACATCAACGTAAAGTGGAAGATCGATGGGTCGGAACGG
CAAAATGGAGTTCTGAATAGCTGGACAGATCAGGATTCTAAGGATTCTACATACTCTATGAGTA
GTACTCTCACACTGACAAAAGATGAGTATGAACGTCATAATAGTTATACCTGCGAAGCGACGC
ATAAGACGTCTACTAGTCCGATTGTTAAAAGCTTTAATCGCAATGAGTGTGATTACAAAGATGA
CGATGACAAATAAttaactcgaggctgagcaaagcagactactaataacataaagtctacgccggacgcatcgtggccctagtacgcaagtt
cacgtaaaaagggtaactagaggttgaggtgattttATGAAAAAGAATATCGCATTTCTTCTTGCATCTATGTTCGTT
TTTTCTATTGCTACAAACGCGTACGCTggtTTCACACTCGAAGATTTCGTTGGGGACTGGGAAC
AGACAGCCGCCTACAACCTGGACCAAGTCCTTGAACAGGGAGGTGTGTCCAGTTTGCTGCAG
AATCTCGCCGTGTCCGTAACTCCGATCCAAAGGATTGTCCGGAGCGGTGAAAATGCCCTGAA
GATCGACATCCATGTCATCATCCCGTATGAAGGTCTGAGCGCCGACCAAATGGCCCAGATCGA
AGAGGTGTTTAAGGTGGTGTACCCTGTGGATGATCATCACTTTAAGGTGATCCTGCCCTATGGC
ACACTGGTAATCGACGGGGTTACGCCGAACATGCTGAACTATTTCGGACGGCCGTATGAAGGC
ATCGCCGTGTTCGACGGCAAAAAGATCACTGTAACAGGGACCCTGTGGAACGGCAACAAAAT
TATCGACGAGCGCCTGATCACCCCCGACGGCTCCATGCTGTTCCGAGTAACCATCAACAGCGG
AGGTACTGGTGGGTCGGGAGGTTCTGGCGGCTCTGGAGGAACTCAGGTTCAACTTCAGCAAT
CAGGCGCTGAATTGGTCCGGCCTGGCACAAGTGTCAAAGTATCGTGTAAGGCGAGTGGTTAC
GCTTTTACTAATCACTTGATCGAATGGGTGAACCAGCGTCCCGGACAGGGTCTGGAATGGATT
GGAGTTATTAATCCGGGGTCTGGTGGGACGAAATATAATGAGAAATTTAAAGGAAAAGCCACA
CTGACCGCGGATAAGTCTAGCTCTACGGCCTACATGCAATTAAGCCGTTTAACTTCTGATGATT
CCGCTGTCTATTTTTGCGCCCGCTCTAGCGAATTTATAACCACCGTTGCCGCAGATTACTGGGG
TCAAGGCACTACTCTCACCGTTTCGAGCGCAAAAACCACTCCGCCTTCAGTCTATCCTCTTGC
GCCTGGATCCGCTGCTCAGACTAACTCAATGGTGACGCTCGGGTGTCTGGTTAAGGGATATTT
TCCTGAACCAGTCACGGTGACATGGAATTCAGGTTCTCTGTCATCTGGAGTCCACACTTTCCC
AGCGGTCCTTCAGTCCGATCTTTATACTCTGTCCAGTTCAGTAACAGTTCCTAGTTCGACATGG
CCATCCGAAACAGTGACGTGTAATGTGGCGCATCCTGCCTCATCAACGAAGGTCGACAAGAA
AATCGTTCCTCGTGATTGTGGT gcggccggccctctggttccggtgattttgattatgaaaagatggcaaacgctaataagggggcta
tgaccgaaaatgccgatgaaaacgcgctacagtctgacgctaaaggcaaacttgattctgtcgctactgattacggtgctgctatcgatggtttcattggtga
cgtttccggccttgctaatggaagtggttccggtgattttgctggctctaattcccaaatggctcaagtcggtgacggtgataattcacctttaatgaataatttc
cgtcaatatttaccttccctccctcaatcggttgaatgtcgcccttttgtctttggcgctggtaaaccatatgaattttctattgattgtgacaaaataaacttattcc
gtggtgtctttgcgtttcttttatatgttgccacctttatgtatgtattttctacgtttgctaacatactgcgtaataaggagtcttaa

Figure S8: DNA sequence of LgBiT-HUI phage. Periplasm signal peptides (gray), HUI variable light chain (light blue), LgBiT (pink), GS/T linker (yellow), HUI variable heavy chain (blue), and M13 P3 (black).

atgaaaaagaatatcgcatttcttcttgcatctatgttcgtttttctattgctacaaatgcctatgcaGACATCCAAATGACTCAAAGTCC
TTCGAGTCTTTCTGCTAGCCTGGGTGGACGCGTGACGATTACTTGTAAGGCGTCGCAGGATAT
CAATAAGTACTTGGCATGGTACCAGCACAAACCGGGTAAGGGTCCACGCTTACTCATTCATTAT
ACTTCCACATTGCAACCAGGTATTCCCAGTCGTTTTAGCGGCTCCGGCAGTGGAAGAGACTAT
TCTTTCAGTATTAGTAATTTAGAACCTGAGGATGTCGCCACATACTATTGCTTACAATACGATAG
CCTGCTTAGTTTCGGGGCAGGGACCAAGCTCGAACTGAAGCGCGCAGACGCCGCACCGACT
GTTTCAATATTCCCACCGAGTTCGGAGCAGTTGACCTCAGGTGGAGCTTCTGTAGTGTGTTTC
TTGAACAACTTCTATCCTAAGGACATAAACGTAAAGTGGAAAATTGACGGCAGTGAAAGACA
GAATGGCGTACTTAACTCTTGGACGGACCAAGATTCGAAGGACTCCACTTACAGTATGTCCTC
AACCCTGACTCTGACGAAAGATGAGTACGAACGACACAATTCCTACACATGCGAGGCTACGC
ATAAGACCTCAACGAGTCCGATTGTAAAGTCGTTTAACCGCAATGAGTGTGACTACAAGGAC
GACGACGACAAATAATTAACTCGAGGCTGAGCAAAGCAGACTACTAATAACATAAAGTCTAC
GCCGGACGCATCGTGGCCCTAGTACGCAAGTTCACGTAAAAAGGGTAACTAGAGGTTGAGGT
GATTTTATGAAGAAGAATATCGCTTTCTTATTGGCCAGTATGTTTGTTTTCTCAATCGCAACGAA
CGCTTATGCCggttccGTGACCGGCTACCGCctgTTTGAGGAGATTCTGGGAGGTACTGGTGGGTC
GGGAGGTTCTGGCGGCTCTGGAGGAACTGAGGTTCAGTTAGTGGAGAGTGGTGGCGGCTTAG
TAAAGCCTGGTGGCTCACTGAAGCTTTCCTGCACAGCATCCGGTTTCGCGTTTAGCGACTATG
ATATGTCATGGGTTCGCCAAACGCCAGAGAAGCGATTGGAGTGGGTCGCGTTCATAAGTAACG
GTGGGTACTCTACCTACTACCCTGACACCGTAAAGGGTAGATTTACGATTTCCCGCGATAACGC
AGAGAACACCCTGTACCTGCAGATGTCTTCTCTCAAATCCGAGGATACAGCGATATACTACTGT
GCACGCCAAGGATTACGCTACTTTGACTACTGGGGCCTTGGTACAACACTGACCGTTAGTAGT
GCGAAGACTACCCCACCTTCCGTCTACCCTTTGGCTCCCGGCAGCGCAGCGCAGACTAACTCT
ATGGTGACATTAGGCTGCCTGGTTAAAGGATACTTTCCAGAACCTGTAACCGTCACGTGGAAC
TCTGGGTCGCTTTCGTCAGGCGTGCACACCTTTCCTGCTGTATTGCAATCCGACCTCTACACGC
TGTCTTCTAGCGTTACGGTTCCCAGCTCAACGTGGCCGTCAGAGACAGTGACCTGTAACGTGG
CTCACCCGGCAAGCTCAACCAAAGTTGACAAGAAGATAGTTCCTCGTGACTGTGGGtgcggccgg
ccctctggttccggtgattttgattatgaaaagatggcaaacgctaataagggggctatgaccgaaaatgccgatgaaaacgcgctacagtctgacgctaa
aggcaaacttgattctgtcgctactgattacggtgctgctatcgatggtttcattggtgacgtttccggccttgctaatggaagtggttccggtgattttgctgg
ctctaattcccaaatggctcaagtcggtgacggtgataattcacctttaatgaataatttccgtcaatatttaccttccctccctcaatcggttgaatgtcgccctt
ttgtctttggcgctggtaaaccatatgaattttctattgattgtgacaaaataaacttattccgtggtgtctttgcgtttcttttatatgttgccacctttatgtatgtatt
ttctacgtttgctaacatactgcgtaataaggagtcttaa

Figure S9: DNA sequence of SmBiT114-OXI phage. Periplasm signal peptides (gray), OXI variable light chain (light green), SmBiT114 (purple), GS/T linker (yellow), OXI variable heavy chain (green), and M13 P3 (black).

Table S1: List of DNA oligos used for cloning procedures.

Oligo Name	Oligo Sequence*
HUI phagemid FWD	TAACCATCAACAGCGGTTCCGATTACAAAGATGACGATGACA
HUI phagemid REV	ACGAAATCTTCGAGTGTGAAACACTCATTGCGATTAAAGC
LgBiT insert FWD	GCTTTAATCGCAATGAGTGTTTCACACTCGAAGATTTCGTTGG
LgBiT insert REV	TCATCGTCATCTTTGTAATCGGAACCGCTGTTGATGGTTA
SmBiT99 Q5 Primer FWD	ttgagaaaattagcggttccGACTACAAGGACGACG
SmBiT99 Q5 Primer REV	acaggcggtagccggtcacACACTCATTGCGGTTAAAC
SmBiT114 Q5 Primer FWD	gaggagattctgtctggttccGACTACAAGGACGACG
SmBiT114 Q5 Primer REV	aaacaggcggtagccggtcacACACTCATTGCGGTTAAAC
Linker Q5 Primer for LgBiT-HUI FWD	gttctggcggctctggaggaactCAGGTTCAACTTCAGC
Linker Q5 Primer for LgBiT-HUI REV	ctcccgacccaccagtacctccGCTGTTGATGGTTACTC
Linker Q5 Primer for SmBiT-OXI FWD	gttctggcggctctggaggaactGAGGTTCAGTTAGTGGAG
Linker Q5 Primer for SmBiT-OXI REV	ctcccgacccaccagtacctccGCTAATTTTCTCAAACAGG
